# Marijuana use and inpatient outcomes among hospitalized patients: analysis of the nationwide inpatient sample database

**DOI:** 10.1002/cam4.968

**Published:** 2016-11-28

**Authors:** Neomi Vin‐Raviv, Tomi Akinyemiju, Qingrui Meng, Swati Sakhuja, Reid Hayward

**Affiliations:** ^1^ University of Northern Colorado Cancer Rehabilitation InstituteGreeleyColorado; ^2^ School of Social WorkCollege of Health and Human SciencesColorado State UniversityFort CollinsColorado; ^3^ Department of EpidemiologyUniversity of Alabama School of Public HealthBirminghamAlabama; ^4^ Comprehensive Cancer CenterUniversity of Alabama School of Public HealthBirminghamAlabama; ^5^ Department of BiostatisticsUniversity of Alabama School of Public HealthBirminghamAlabama; ^6^ School of Sport and Exercise ScienceUniversity of Northern ColoradoGreeleyColorado

**Keywords:** Cancer patients, hospitalized patients, inpatient outcomes, marijuana use

## Abstract

The purpose of this paper is to examine the relationship between marijuana use and health outcomes among hospitalized patients, including those hospitalized with a diagnosis of cancer. A total of 387,608 current marijuana users were identified based on ICD‐9 codes for marijuana use among hospitalized patients in the Nationwide Inpatient Sample database between 2007 and 2011. Logistic regression analysis was performed to determine the association between marijuana use and heart failure, cardiac disease, stroke, and in‐hospital mortality. All models were adjusted for age, gender, race, residential income, insurance, residential region, pain, and number of comorbidities. Among hospitalized patients, marijuana use was associated with a 60% increased odds of stroke (OR: 1.60, 95% CI: 1.44–1.77) compared with non‐users, but significantly reduced odds of heart failure (OR: 0.78, 95% CI: 0.75–0.82), cardiac disease (OR: 0.86, 95% CI: 0.82–0.91), or in‐hospital mortality (OR: 0.41, 95% CI: 0.38–0.44). Among cancer patients, odds of in‐hospital mortality was significantly reduced among marijuana users compared with non‐users (OR: 0.44, 95% CI: 0.35–0.55). Hospitalized marijuana users were more likely to experience a stroke compared with non‐users, but less likely to experience in‐hospital mortality. Prospective studies will be needed to better characterize the health effects of marijuana use, especially among older, sicker, and/or hospitalized patients. In the meantime, conversations regarding marijuana use/misuse may be warranted in the clinical setting in order for patients and healthcare providers to adequately weigh the anticipated benefits of marijuana use with potentially significant health risks.

## Introduction

In the United State, medical marijuana is legal in 28 states, the District of Columbia, Guam and Puerto Rico. While recreational use of marijuana is legal now legal in 7 states and in the District of Columbia 1. Cancer is a qualifying medical condition in every state that has approved marijuana for medical use 2. Decriminalization of marijuana use and possession in some of these states has led to more widespread use 3. In 2015, the number of cancer registrants in the Colorado Medical Marijuana Registry grew rapidly — from 908 in December 2009 to 3918 in December 2015 4. Increasing numbers of Western countries have legalized the use of medical marijuana, and there have been marked increases in the use of synthetic or plant‐derived cannabinoids (e.g., dronabinol, nabilone) for the treatment of a wide range of cancer‐related symptoms 2, 5, 6, 7 such as anorexia, nausea/vomiting, spasticity, and pain. Yet, the efficacy and use of marijuana in oncology settings is a subject of complex and controversial debate 8. Emerging evidence supports the short‐term benefits of marijuana use for symptom relief including nausea and vomiting associated with cancer chemotherapy 9. However, most of the existing data are based on animal models, small clinical trials and/or are outdated 10. A recent review by Whiting and colleagues found low‐quality evidence suggesting that cannabinoids were associated with improvements in nausea and vomiting due to chemotherapy 11.

The limited data on effectiveness contrast with accumulating data suggesting a number of harmful medical effects of marijuana on users 9. Recreational marijuana use has been linked to several adverse health outcomes including addiction, impaired cognition, and mental illness 9, 12. Additionally, recent evidence suggests that recreational marijuana use may be associated with both cardiovascular and cerebrovascular events 13, 14, 15, and frequent use has been associated with increased risk of myocardial infarction 16, 17. Research studies have also demonstrated that recreational marijuana uses is associated with subarachnoid hemorrhage, intracerebral hemorrhage, and acute ischemic stroke 18, 19. A recent review examining medical use of the three approved cannabis‐based medications and ingested marijuana found that adverse effects included a major increase in cardiovascular‐related adverse effects such as tachycardia, orthostatic hypotension, hypertension, palpitations, paroxysmal atrial fibrillation, and peripheral vasodilation 20. The effect of marijuana use on short‐ and long‐term health outcomes in cancer patients is less clear. In oncology settings, most studies to date have focused specifically on marijuana use among advanced cancer patients 6, 21. Therefore, the magnitude of side effects due to marijuana could not be accurately assessed. More research is needed to elucidate the potential adverse and beneficial effects of marijuana use on health outcomes 10, 20.

In view of the rapidly increased use of marijuana among cancer patients who are already at significantly increased risk for treatment‐related cardiovascular and pulmonary toxicities 22, 23, 24, 25, it is important to examine the relationship between marijuana use and other health outcomes. Thus, the purpose of this study was to assess the prevalence of marijuana use among hospitalized patients with and without a primary cancer diagnosis, and its association with health outcomes. By utilizing data from the large Nationwide Inpatient Sample database, we are able to examine relatively rare events, using clinical data to document diagnoses of cancer and marijuana uses.

## Methods

### Data source

Data for this study were obtained from the Health Cost and Utilization Project Nationwide Inpatient Sample (HCUP‐NIS) inpatient database. The HCUP‐NIS is a large all‐payer inpatient care database which includes over seven million hospitals stays and covers over 1000 hospitals in the US 26. Diagnostic and procedural information in the NIS are identified using the International Classification of Diseases, Ninth Edition, Clinical Modification (ICD‐9‐CM). This database contains clinical variables on all diagnoses and procedures during each hospital admission and nonclinical variables including median household income in the patient's zip code, rural/urban residence, and expected payment source. The HCUP‐NIS database is widely considered the most valid and reliable source of epidemiological data on in‐patient care and outcomes in the U.S. More information on HCUP‐NIS can be obtained at https://www.hcup-us.ahrq.gov/nisoverview.jsp. The HCUP‐NIS data used in this study represents deidentified, publicly available secondary data, and therefore the study was considered exempt from ethical review by the Institutional Review Board of the University of Alabama at Birmingham.

### Sample selection

In order to evaluate the relationships between marijuana use and clinical outcomes among hospitalized patients in the NIS database between 2007 and 2011, a sample of current marijuana users were identified based on ICD codes for cannabis dependence use (ICD‐9 codes: 304.30‐2) and cannabis nondependence abuse (ICD‐9 codes: 305.20‐2). Our definition of marijuana use is similar to the one used by Rumalla et al. in the NIS database 18, 19. Since there is no specific ICD‐9‐CM code for medical marijuana use, we made the assumption that patients with clinical diagnoses of cannabis abuse represent patients with recreational marijuana use. Several studies show that a large share of medicinal users also use cannabis recreationally 27, 28, and a recent study estimated that approximately 86% of people who report ever using cannabis for medicinal purposes also use it recreationally 29. Therefore, in this paper, ICD‐9‐CM codes for cannabis abuse were used to represent recreational marijuana use. We further evaluated the association between marijuana use among hospitalized patients that were secondary to the cancer diagnosis (ICD‐9‐CM codes for cancer 140‐149, 150‐159, 160‐165, 170‐176, 179‐189, 190‐199, 200‐209).

### Outcome measures

Our primary outcomes of interest included heart failure (ICD‐9 codes: 428.0‐1, 428.20‐3, 428.30‐3, 428.40‐3, 428.9), cardiac disease (ICD‐9 codes: 427.0‐2, 427.31‐2, 427.41‐2, 427.5, 427.60‐1, 427.69, 427.81, 427.89, 427.9), stroke (ICD‐9 codes: 433.01, 433.11, 433.21, 433.31, 433.81, 433.91, 434.01, 434.11, 434.9, 436), and in‐hospital mortality. In‐hospital mortality was defined by deaths occurring during hospitalization, and was examined among all patients as well as patients with a primary diagnosis of cancer.

### Individual variables

Other covariates used in the analysis included race/ethnicity (White, Black, Hispanic, Other), age (<40, 40–50, 50–60, 60–70, and >70 years), gender, and insurance type (classified into Medicaid, Medicare, Private including Blue Cross, commercial carriers, private HMOs, PPOs, and self‐insured and Others containing Worker's Compensation, Title V, and other government programs) (1). Residential income was categorized into quartiles ranging from the lowest income to the highest income based on median household income at the zip‐code level. Residential region was classified into large metropolitan areas (metropolitan areas with 1 million residents or more), small metropolitan areas (metropolitan areas with less than 1 million residents), micropolitan areas (nonmetropolitan areas adjacent to metropolitan areas) and nonmetropolitan or micropolitan areas (noncore areas with or without own town) according to the 2003 version of the Urban Influence Codes (2). Comorbidities were identified using ICD‐9 codes and used to calculate a modified Deyo‐Charlson index 30. The original 17‐item index is a validated measure of comorbidity for administrative data 31, 32, 33, however, the modified Deyo‐Charlson index has been used extensively in the NIS database 34, 35, 36, 37. In the current analysis, the following comorbid conditions were included: Dementia, diabetes mellitus with and without complication, chronic pulmonary disease, rheumatic disease, peptic ulcer disease, mild liver disease, hemiplegia or paraplegia, renal disease, moderate or severe liver disease, and HIV/AIDS. Other conditions were excluded as they were part of our study outcomes, for example, myocardial infarction, congestive heart failure, peripheral vascular disease, and cerebrovascular disease. The presence of each condition within each patient was identified, and a single comorbidity score was created as the sum of the number of conditions per patient and categorized as 0, 1, or ≥2. 30. In addition, due to increasing number of studies demonstrating that many individuals (45–80%) seek medical marijuana for reasons related to pain relief 38, 39, 40, we examined pain (ICD‐9 code: 338.0‐1, 338.11‐2, 338.18‐9, 338.2, 338.21‐2, 338.28‐9, 338.3‐4) as a covariate in the analysis.

### Statistical analysis

We conducted descriptive statistics to examine differences in the distribution of marijuana use by race/ethnicity and other socio‐demographic variables using chi‐squared tests. Two study populations were analyzed in this study; all patients regardless of diagnosis, as well as patients with a primary diagnosis of cancer. Multivariable logistic regression analysis was performed for each study population to determine the association between marijuana use and heart failure, cardiac disease, stroke, and in‐hospital mortality adjusting for race/ethnicity, age, gender, residential income, insurance type, residential region, and comorbidities Results are presented as odds ratios and 95% confidence intervals, and all statistical analyses were conducted in SAS 9.4.

## Results

This study included a total of approximately 3.9 million patients; of those, 387,608 had a diagnosis code for marijuana use. Marijuana users were younger compared with non‐users (65% vs. 37% <40 years), more likely to be male (62% vs. 42%), black (31% vs. 14%), and belong to the lowest residential income quartile (40% vs. 29%). They were also less likely to have private insurance (23% vs. 34%), and more likely to have no comorbid condition on admission (78% vs. 71%) compared with non‐users (Table 1). Marijuana users were less likely to have a diagnosis of cancer (1.2% vs. 5.5%) compared with non‐users, but among cancer patients, those with lymphatic cancer were more likely to also have a diagnosis code for marijuana use (25% vs. 22%). All *P* < 0.001.

**Table 1:** The distribution of Socio‐Demographic characteristics by marijuana use, Nationwide Inpatient Sample, 2007–2011

Study characteristics	Marijuana use *N* (%)		*P‐*value
	Yes (*N* = 387608)	No (*N* = 39448981)	
Age, years			<0.0001
<40	252219 (65.1)	14510000 (36.8)	
40 to <50	73686 (19.0)	3723768 (9.4)	
50 to <60	49498 (12.8)	4913174 (12.5)	
60 to <70	10526 (2.7)	5261754 (13.3)	
≥70	1679 (1.2)	11040000 (28.0)	
Gender			<0.0001
Female	146496 (37.8)	23020000 (58.5)	
Male	240785 (62.2)	16330000 (41.5)	
Race			<0.0001
White	174280 (55.0)	21640000 (66.1)	
Black	97122 (30.7)	4705251 (14.4)	
Hispanic	28918 (9.1)	4132190 (12.6)	
Other	16374 (5.2)	2273962 (6.9)	
Residential income			<0.0001
First quartile‐lowest	149512 (40.4)	11130000 (28.9)	
Second quartile	94042 (25.4)	10000000 (26.0)	
Third quartile	75056 (20.3)	9188328 (23.9)	
Fourth quartile‐highest	51672 (14.0)	8166927 (21.2)	
Insurance type			<0.0001
Medicare	52124 (13.5)	14890000 (37.8)	
Medicaid	138193 (35.8)	7764768 (19.7)	
Private	88180 (22.9)	13200000 (33.5)	
Other	107391 (27.8)	3503379 (8.9)	
Residential region			<0.0001
Large metro	217056 (61.1)	20750000 (58.3)	
Small metro	100806 (28.4)	10490000 (29.5)	
Micropolitan	37375 (10.5)	4342303 (12.2)	
Comorbidities			<0.0001
0	304156 (78.5)	28070000 (71.2)	
1	73141 (18.9)	8745045 (22.2)	
≥2	10311 (2.7)	2629913 (6.7)	
Any cancer			<0.0001
Yes	4814 (1.2)	2177046 (5.5)	
No	382794 (98.8)	37270000 (94.5)	
Cancer type			<0.0001
Breast cancer	177 (3.7)	119992 (5.5)	
Colorectal cancer	258 (5.4)	127805 (5.9)	
Lung cancer	537 (11.2)	300889 (13.8)	
Lymphatic cancer	1206 (25.1)	476195 (21.9)	
Other	2636 (54.8)	1152165 (52.9)	
Heart failure			<0.0001
Yes	3146 (0.8)	990434 (2.5)	
No	384462 (99.2)	38460000 (97.5)	
Cardiac			<0.0001
Yes	2812 (0.7)	786550 (2.0)	
No	384796 (99.3)	38660000 (98.0)	
Stroke			<0.0001
Yes	594 (0.2)	105363 (0.3)	
No	387014 (99.8)	39340000 (99.7)	
Pain			<0.0001
Yes	285 (0.1)	57223 (0.2)	
No	387323 (99.9)	39390000 (99.8)	
Died			<0.0001
Yes	1379 (0.4)	769308 (2.0)	
No	385796 (99.6)	38650000 (98.0)	

*P*‐values from chi‐squared tests assessing for significant differences in study characteristics by marijuana use status.

Using a multivariable adjusted analysis among all hospitalized patients, marijuana use was associated with significantly reduced odds of heart failure (OR: 0.78, 95% CI: 0.75–0.82), cardiac disease (OR: 0.86, 95% CI: 0.82–0.91), and in‐hospital mortality (OR: 0.41, 95% CI: 0.38–0.44). However, marijuana use was associated with a significant 60% increased odds of stroke (OR: 1.60, 95% CI: 1.44–1.77) compared with non‐users (Table 2). Among cancer patients (Table 3), marijuana use was associated with slightly increased, but not statistically significant odds of heart failure (OR: 1.08, 95% CI: 0.74–1.56), cardiac disease (OR: 1.13, 95% CI: 0.76–1.69), and reduced odds of stroke (OR: 0.25, 95% CI: 0.04–1.78). However, odds of in‐hospital mortality was significantly reduced among marijuana users compared with non‐users (OR: 0.44, 95% CI: 0.35–0.55). All models were adjusted for age, gender, race, residential income, insurance, residential region, and number of comorbidities. The association between marijuana use and in‐hospital mortality among cancer patients remained consistent when stratified by pain status, and adjusting for pain in the statistical models did not attenuate the association between marijuana use and in‐hospital mortality (data not shown).

**Table 2:** Multivariable adjusted associations between marijuana use^c^ and hospitalization outcomes among ALL patients, Nationwide Inpatient Sample 2007–2011^a^

	Heart failureOR (95% CI)*N* = 993580	CardiacOR (95% CI)*N* = 789362	StrokeOR (95% CI)*N* = 105957	Death^b^OR (95% CI)*N* = 770687
Marijuana use				
Yes	0.78 (0.75, 0.82)	0.86 (0.82, 0.91)	1.60 (1.44, 1.77)	0.41 (0.38, 0.44)
No	Ref	Ref	Ref	Ref
Age, years				
<40	0.05 (0.05, 0.05)	0.06 (0.06, 0.06)	0.03 (0.02, 0.03)	0.68 (0.66, 0.71)
40 to <50	0.29 (0.29, 0.30)	0.31 (0.30, 0.31)	0.23 (0.23, 0.25)	0.21 (0.21, 0.22)
50 to <60	0.46 (0.45, 0.46)	0.50 (0.49, 0.50)	0.42 (0.41, 0.43)	0.36 (0.35, 0.36)
60 to <70	0.61 (0.61, 0.62)	0.74 (0.74, 0.75)	0.65 (0.63, 0.66)	0.52 (0.51, 0.52)
≥70	Ref	Ref	Ref	Ref
Gender				
Female	0.85 (0.84, 0.85)	0.82 (0.82, 0.83)	0.84 (0.83, 0.85)	0.82 (0.82, 0.83)
Male	Ref	Ref	Ref	Ref
Race				
Black	1.65 (1.63, 1.66)	0.75 (0.75, 0.76)	1.05 (1.02, 1.07)	1.06 (1.05, 1.07)
Hispanic	1.14 (1.13, 1.15)	0.73 (0.73, 0.74)	0.83 (0.80, 0.85)	0.98 (0.97, 0.99)
Other	1.02 (1.01, 1.03)	0.83 (0.82, 0.85)	1.07 (1.04,1.11)	1.20 (1.19, 1.22)
White	Ref	Ref	Ref	Ref
Residential income				
First quartile‐lowest	1.16 (1.15, 1.17)	0.93 (0.93, 0.94)	0.87 (0.85, 0.89)	1.02 (1.01, 1.03)
Second quartile	1.12 (1.11, 1.13)	0.97 (0.96, 0.94)	0.90 (0.89, 0.92)	0.98 (0.97, 0.99)
Third quartile	1.06 (1.06, 1.07)	0.99 (0.98, 0.99)	0.96 (0.94, 0.97)	0.97 (0.96, 0.98)
Fourth quartile‐ highest	Ref	Ref	Ref	Ref
Insurance type				
Medicaid	1.62 (1.60, 1.63)	0.69 (0.68, 0.70)	1.04 (1.01, 1.08)	1.30 (1.28, 1.32)
Medicare	1.41 (1.39, 1.42)	0.88 (0.87, 0.89)	0.88 (0.86, 0.90)	0.95 (0.94, 0.95)
Other	1.42 (1.41, 1.44)	0.81 (0.80, 0.83)	1.27 (1.23, 1.32)	1.51 (1.49, 1.53)
Private	Ref	Ref	Ref	Ref
Residential region				
Small metro	0.99 (0.99, 1.00)	1.04 (1.03, 1.04)	1.04 (1.02, 1.06)	1.00 (0.99, 1.01)
Micropolitan	1.00 (0.99, 1.01)	1.07 (1.06, 1.08)	0.95 (0.93, 0.98)	1.03 (1.02, 1.04)
Large metro	Ref	Ref	Ref	Ref
Pain				
Yes	—	—	—	1.73 (1.64, 1.83)
No				Ref
Comorbidities				
0	Ref	Ref	Ref	Ref
1	2.45 (2.43, 2.46)	0.92 (0.91, 0.92)	1.15 (1.14, 1.17)	1.47 (1.46, 1.48)
≥2	4.31 (4.29, 4.34)	0.84 (0.83, 0.84)	1.14 (1.12, 1.17)	1.86 (1.85, 1.88)

a Models adjusted for age, gender, race, residential income, insurance, region, pain, and number of comorbidities.

b Deaths refer to mortality occurring during admission.

c Current marijuana use defined based on ICD‐9 codes based on dependence use of cannabis.

**Table 3:** Multivariable adjusted associations between marijuana use^c^ and hospitalization outcomes among CANCER patients, Nationwide Inpatient Sample 2007–2011^a^

	Heart failureOR (95% CI)*N* = 40687	CardiacOR (95% CI)*N* = 33059	StrokeOR (95% CI)*N* = 5051	Death^b^OR (95% CI)*N* = 147260
Marijuana use				
Yes	1.08 (0.74, 1.56)	1.13 (0.76, 1.69)	0.25 (0.04, 1.78)	0.44 (0.35, 0.55)
No	Ref	Ref	Ref	Ref
Age, years				
<40	0.05 (0.04, 0.05)	0.06 (0.06, 0.06)	0.02 (0.02, 0.02)	0.09 (0.09, 0.09)
40 to <50	0.21 (0.19, 0.23)	0.21 (0.19, 0.23)	0.31 (0.26, 0.38)	0.53 (0.51, 0.55)
50 to <60	0.31 (0.30, 0.33)	0.38 (0.36, 0.40)	0.45 (0.40, 0.51)	0.66 (0.65, 0.68)
60 to <70	0.48 (0.46, 0.49)	0.64 (0.62, 0.67)	0.64 (0.59, 0.70)	0.79 (0.78, 0.80)
≥70	Ref	Ref	Ref	Ref
Gender				
Female	0.94 (0.92, 0.96)	0.80(0.78, 0.82)	1.03 (0.96, 1.10)	0.91 (0.90, 0.93)
Male	Ref	Ref	Ref	Ref
Race				
Black	1.31 (1.26, 1.35)	0.77 (0.74, 0.81)	0.89 (0.80, 0.99)	1.13 (1.11, 1.15)
Hispanic	0.98 (0.94, 1.04)	0.62 (0.58, 0.66)	0.72 (0.62, 0.84)	1.01 (0.98, 1.04)
Other	0.83 (0.78, 0.88)	0.73 (0.68, 0.78)	0.90(0.77, 1.05)	1.29(1.25, 1.32)
White	Ref	Ref	Ref	Ref
Residential income				
First quartile‐lowest	1.04 (1.01, 1.08)	1.01 (0.97, 1.05)	0.79 (0.72, 0.88)	1.00 (0.98, 1.02)
Second quartile	1.06 (1.03, 1.10)	1.02 (0.99, 1.06)	0.83 (0.76, 0.92)	0.96 (0.94, 0.98)
Third quartile	1.06 (1.02, 1.09)	1.05 (1.02, 1.09)	0.94 (0.86, 1.02)	0.96 (0.94, 0.98)
Fourth quartile‐highest	Ref	Ref	Ref	Ref
Insurance type				
Medicaid	1.13 (1.06, 1.21)	0.89 (0.83, 0.96)	1.09 (0.93, 1.28)	1.03 (1.00, 1.06)
Medicare	1.39 (1.33, 1.44)	1.14 (1.10, 1.19)	0.99 (0.90, 1.09)	0.80 (0.78, 0.81)
Other	1.04 (0.96, 1.13)	0.83 (0.77, 0.91)	1.23 (1.03, 1.47)	1.52 (1.48, 1.57)
Private	Ref	Ref	Ref	Ref
Residential region				
Small metro	1.00 (0.98, 1.03)	1.02 (0.99, 1.05)	1.06 (0.98, 1.15)	1.02 (1.01, 1.04)
Micropolitan	1.01 (0.97, 1.05)	1.04 (0.99, 1.08)	1.08 (0.96, 1.21)	1.06 (1.04, 1.09)
Large metro	Ref	Ref	Ref	Ref
Pain				
Yes	—	–	—	1.25 (1.18, 1.32)
No				Ref
Comorbidities				
0	Ref	Ref	Ref	Ref
1	2.35 (2.28, 2.41)	1.19 (1.16, 1.22)	1.26 (1.18, 1.36)	1.31 (1.29, 1.33)
≥2	4.34 (4.20, 4.48)	1.22 (1.17, 1.27)	1.48 (1.34, 1.65)	1.43 (1.40, 1.46)

a Models adjusted for age, gender, race, residential income, insurance, region, pain, and number of comorbidities.

b Deaths refer to mortality occurring during admission.

c Current Marijuana use defined based on ICD‐9 codes based on dependence use of cannabis.

## Discussion

Marijuana is becoming increasingly available to the general population given the rapidly changing landscape surrounding marijuana use and possession policies across the US. There is significant interest in examining the health effects of marijuana among adults in general, but more importantly among those with health conditions or chronic diseases. In this study, we examined the relationships between marijuana use and health outcomes among hospitalized patients, including hospitalized patients with and without a diagnosis of cancer using ICD‐9 codes. We observed that among hospitalized patients, marijuana use was associated with significantly reduced odds of heart failure and cardiac disease compared with non‐users. However, marijuana use was associated with significantly increased odds of stroke in all hospitalized patients compared with non‐users. Among cancer patients, there was a positive but nonsignificant association between marijuana use and heart failure and cardiac disease, and an inverse but nonsignificant association between marijuana use and stroke. The odds of in‐hospital mortality were significantly reduced among marijuana users compared with non‐users overall, and among cancer patients.

The vast majority of studies examining the potential adverse effects of marijuana use on health have assessed cardiac‐related conditions, and several have documented increased risks of cerebrovascular events associated with marijuana use, ranging from transient ischemic events to strokes 18, 19, 41, 42. However, other studies have failed to observe any increased risk of adverse health outcomes associated with marijuana use 43, 44. Conflicting results may be due to major differences in study population, underlying health status, source of data on marijuana use (e.g., self‐reported vs. clinical data), or reason for marijuana use (medical vs. recreational). For instance, a previous study comparing the incidence of acute ischemic stroke between marijuana users and non‐users focused only on young patients (age groups 15–54) 19, while other studies have examined stroke outcomes in elderly individuals, which are usually thromboembolic or related to carotid artery disease 45 in contrast with the intracranial vascular etiology in younger patients 46, 47, 48. Therefore, the influence of marijuana use on cardiac outcomes may vary by age due to differing etiological factors. Fewer studies have focused on homogenous study populations using objective measures to assess marijuana use and health outcomes. Our study addresses some of these limitations by focusing on hospitalized patient's ages 40 years and older, using ICD‐9 claims data to assess both marijuana use and health outcomes in a cross‐sectional setting. Consistent with previous findings by Rumalla et al. 19, we observed significantly higher odds of strokes among marijuana users compared with non‐users among adult hospitalized patients, however, this association was not significant among cancer patients.

We also observed that odds of in‐hospital mortality were significantly reduced among marijuana users compared with non‐users in all hospitalized patients as well as cancer patients. This finding stands in apparent contrast to previous reports suggesting that marijuana use can serve as a trigger for acute coronary syndromes and marijuana‐related vascular complications that are associated with elevated mortality 17, 49. Rumalla et al. 19 observed that marijuana users were more likely to use other illicit substances compared with non‐users although they had less overall comorbidities. This suggests that complications arising from the use of multiple substances may lead to adverse health outcomes. Other studies where marijuana use was linked with adverse health outcomes such as myocardial infarction and death also reported that patients tended to be younger and have additional risk factors, such as cocaine, tobacco, and alcohol use 17, 50. For example, an almost fivefold increased risk for myocardial infarction within the hour after marijuana use was reported by Mittleman and colleagues, although the risk decreased after the initial hour post‐use 50. Similarly, a 4.2‐fold increase in mortality rate was observed in marijuana users compared with non‐users following myocardial infarction 16. The coronary effects of marijuana exposure have also been linked to cardiac arrhythmias and/or sudden cardiac death, although these appear to be relatively rare events 51, 52.

Our finding of an inverse association between marijuana use and in‐hospital mortality deserves further study. It is possible that advancements in medical care, including rapid diagnosis and high‐quality treatment that has led to a substantial improvement in hospitalization outcomes for cardiovascular disease and reduction in adverse events 53, 54 regardless of underlying risk factors or exposure such as marijuana use. It is also possible that among older, sicker adults, marijuana use not accompanied by other illicit or harmful risk factors such as cocaine and/or alcohol use 55, but is associated with some health benefits such as reduced risk of adverse cardiac outcomes. However, in this cross‐sectional study design, it is impossible to definitively rule out confounding or to conclude that marijuana use reduces the risk of adverse health outcomes. Larger prospective studies with objective measures of marijuana use and health outcomes will be needed to better examine these associations. Nevertheless, these findings provide information suggesting that marijuana use is negatively associated with certain health outcomes that may be important for older, sicker population groups. Pain management in this population subgroup is often the main reason for medical marijuana use 27, 56, however, we observed similar associations between marijuana use and health outcomes regardless of clinical diagnosis of pain although there is no straightforward and reliable method to identify chronic pain using administrative databases 57.

The presented results are subject to certain limitations. First, the analyses were based on a database of inpatient hospitalizations and therefore only health outcomes that were captured during inpatient admissions were included in this study. Second, we evaluate the relationships between a clinical diagnosis of marijuana use with clinical outcomes using the NIS database between 2007 and 2011. The NIS databases are based on the ICD‐9‐CM coding system, which has been used in the US since 1979. To comply with recent rules on adoption of the ICD‐10‐CM, the NIS databases also replaced the current ICD‐9‐CM coding system beginning from October 1, 2015 58. Third, our operational definition of marijuana use relied exclusively on ICD‐9‐CM codes documented in the NIS databases. As with any administrative claims database, there is a chance of misclassification and underclassification of drug use using secondary ICD‐9‐CM codes as it is often self‐reported 59. Although diagnostic inaccuracy may also be a manifestation of the ICD‐9‐CM diagnosis system that categorize participants solely on the basis of dependency status, other studies have also accurately identified marijuana use ICD‐9‐CM diagnosis codes 18, 19. Fourth, the extent to which cannabis use represents medical or recreational use is unknown. No formal coding exists to specify the specific indication, dose, or timing of use. Therefore, we are unable to directly assess causality or dose–response mechanisms in this analysis due to the cross‐sectional nature of the dataset.

There are also several strengths of this analysis. First, there is very little direct evidence regarding the association between marijuana use and health outcomes among older, sicker adults in the US. Second, the associations between marijuana use and health outcomes were assessed using clinical claims data in a nationally representative dataset, with detailed adjustment for multiple confounders. Our study adds to the growing body of evidence suggesting that high‐quality, prospective studies of marijuana consumption and health outcomes among adults with multiple chronic conditions are warranted to better elucidate the mechanisms underlying the increased risk of stroke, and to determine whether the inverse association with mortality remains consistent. As the use of both medical and recreational marijuana becomes increasingly prevalent for pain management or other purposes, knowledge and awareness among health care professionals is essential to educate patients about the appropriate use of marijuana. Both health care providers and patients will need to carefully consider the anticipated benefits in light of potentially significant health risks.

## Conflict of Interest

None declared.
